# Erratum to: An approach to the research on ion and water properties in the interphase between the plasma membrane and bulk extracellular solution

**DOI:** 10.1007/s12576-017-0572-6

**Published:** 2017-10-04

**Authors:** Hiroshi Hibino, Madoka Takai, Hidenori Noguchi, Seishiro Sawamura, Yasufumi Takahashi, Hideki Sakai, Hitoshi Shiku

**Affiliations:** 1 Department of Molecular Physiology, Niigata University School of Medicine, 1-757 Asahimachi-dori, Chuo-ku, Niigata, Niigata 951-8510 Japan; 2 Center for Transdisciplinary Research, Niigata University, Niigata, Niigata 950-2181 Japan; 3 AMED-CREST, AMED, Niigata, Japan; 4 Department of Bioengineering, School of Engineering, The University of Tokyo, Tokyo, Japan; 5 International Center for Materials Nanoarchitectonics and Global Research Center for Environment and Energy Based on Nanomaterials Science, National Institute for Materials Science, Tsukuba, Ibaraki Japan; 6 Division of Electrical Engineering and Computer Science, Kanazawa University, Kakuma-machi, Kanazawa, 920-1192 Japan; 7 Precursory Research for Embryonic Science and Technology (PRESTO), Japan Science and Technology Agency (JST), Saitama, 332-0012 Japan; 8 Department of Pharmaceutical Physiology, Graduate School of Medicine and Pharmaceutical Sciences, University of Toyama, Toyama, Japan; 9 Graduate School of Engineering, Tohoku University, Sendai, 980-8579 Japan

## Erratum to: J Physiol Sci (2017) 67:439–445 DOI 10.1007/s12576-017-0530-3

The article “An approach to the research on ion and water properties in the interphase between the plasma membrane and bulk extracellular solution”, written by Hiroshi Hibino, Madoka Takai, Hidenori Noguchi, Seishiro Sawamura, Yasufumi Takahashi, Hideki Sakai and Hitoshi Shiku, was originally published Online First without open access. After publication in volume 67, issue 4, pages 439–445 the author decided to opt for Open Choice and to make the article an open access publication. Therefore, the copyright of the article has been changed to © The Author(s) 2017 and the article is forthwith distributed under the terms of the Creative Commons Attribution 4.0 International License (http://creativecommons.org/licenses/by/4.0/), which permits use, duplication, adaptation, distribution and reproduction in any medium or format, as long as you give appropriate credit to the original author(s) and the source, provide a link to the Creative Commons license, and indicate if changes were made.

